# The frequency and validity of self-reported diagnosis of Parkinson's Disease in the UK elderly: MRC CFAS cohort

**DOI:** 10.1186/1471-2377-6-29

**Published:** 2006-08-22

**Authors:** Thomas Foltynie, Fiona E Matthews, Lianna Ishihara, Carol Brayne, MRC CFAS

**Affiliations:** 1 Cambridge Centre for Brain Repair, University of Cambridge, Forvie Site, Robinson Way, Cambridge, CB2 2PY, UK; 2 MRC Biostatistics Unit, Institute of Public Health, University of Cambridge, Forvie Site, Robinson Way, Cambridge, CB2 2SR, UK; 3 Dept. Public Health and Primary Care, Institute of Public Health, University of Cambridge, Forvie Site, Robinson Way, Cambridge, CB2 2SR, UK

## Abstract

**Background:**

Estimates of the incidence and prevalence of chronic diseases can be made using established cohort studies but these estimates may have lower reliability if based purely on self-reported diagnosis.

**Methods:**

The MRC Cognitive Function & Ageing Study (MRC CFAS) has collected longitudinal data from a population-based random sample of 13004 individuals over the age of 65 years from 5 centres within the UK. Participants were asked at baseline and after a two-year follow-up whether they had received a diagnosis of Parkinson's disease. Our aim was to make estimates of the incidence and prevalence of PD using self-reporting, and then investigate the validity of self-reported diagnosis using other data sources where available, namely death certification and neuropathological examination.

**Results:**

The self-reported prevalence of Parkinson's disease (PD) amongst these individuals increases with age from 0.7% (95%CI 0.5–0.9) for 65–75, 1.4% (95%CI 1.0–1.7) for 75–85, and 1.6% (95%CI 1.0–2.3) for 85+ age groups respectively. The overall incidence of self reported PD in this cohort was 200/100,000 per year (95%CI 144–278). Only 40% of the deceased individuals reporting prevalent PD and 35% of those reporting incident PD had diagnoses of PD recorded on their death certificates. Neuropathological examination of individuals reporting PD also showed typical PD changes in only 40%, with the remainder showing basal ganglia pathologies causing parkinsonism rather than true PD pathology.

**Conclusion:**

Self-reporting of PD status may be used as a screening tool to identify patients for epidemiological study, but inevitably identifies a heterogeneous group of movement disorders patients. Within this group, age, male sex, a family history of PD and reduced cigarette smoking appear to act as independent risk factors for self-reported PD.

## Background

A review of worldwide incidence figures for Parkinson's disease (PD) was published in 2003[1]. This article correctly points out the major difficulties in comparing figures from one study to the next- namely differing methods of case ascertainment, differing inclusion and exclusion criteria and lack of follow up or histology to confirm diagnoses. Age-specific rates for groups of patients over 65 years using similar methodologies find variable incidence rates ranging from 29[2] to 222[3] per 100,000 per year. Worldwide prevalence rates also vary due to differing study methodologies, and comparisons are made even more difficult due to international differences in general population survival[4].

Studies that attempt to identify unrecognised cases within the population using screening methods find higher incidence and prevalence rates than studies using medical records or death certificates. Studies of older cohorts identify much larger total numbers of PD patients, and generate more stable estimates of the disease frequency among those people most at risk. Age-specific rates established using similar methodologies among older groups of patients find prevalence rates varying from a low of 198 per 100 000 in >50 year age group in China[5]. rising to 600 per 100,000 in 65–69 age groups, through to 3500 per 100 000 in 85–89 year age groups in Europe[6].

The MRC Cognitive Function in Ageing Study- CFAS is a large prospective cohort study established in 1991 to examine the frequency of cognitive dysfunction and evaluate possible risk factors for disturbed cognition amongst the population over the age of 65 in the UK. Details of the study have been previously published elsewhere. [7]. This study has not sought to identify participants with unrecognised symptoms of PD, but includes self-reported PD status for all individuals. In this analysis, we present age and sex specific figures for the self reported prevalence and incidence of PD within CFAS participants over the first 2 years of follow up. We have also calculated prevalence and incidence rates following standardisation to the 1991 European population age structure.

Self-reporting of PD status has lower precision for the diagnosis than clinical assessment by a movement disorders clinician[8], which in turn has a slightly lower precision for the diagnosis than neuropathological examination[9]. In this study we have compared the frequency of PD based on self-report with the frequency of PD recorded on death certificates, and in a sample of our patients we have been able to explore the accuracy of self-reported PD status using post mortem neuropathological diagnoses.

## Methods

**Figure 1:**
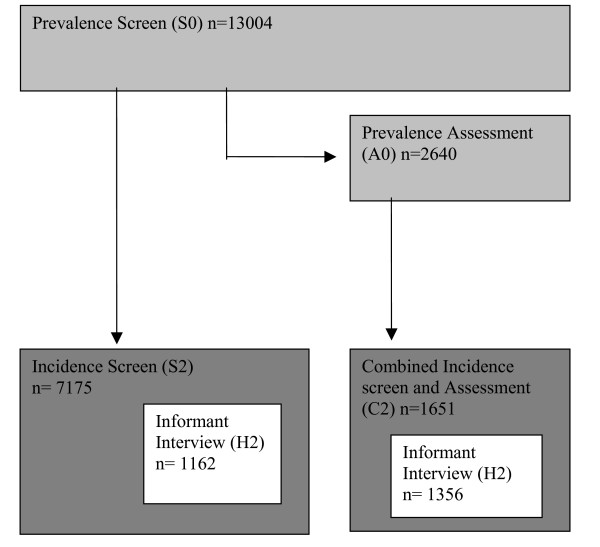
Schematic figure representing the design of the initial phases of the CFAS study.

At each of 5 centres within the UK (Cambridgeshire, North Wales (Gwynedd), Newcastle, Nottingham and Oxford), random samples of approximately 2500 people aged 65 years and above were recruited to this study (N = 13,004 total participants). A structured interview was performed among all these individuals at baseline (S0), including a question on PD diagnosis-see Figure 1. In addition personal historical information was obtained including quantitative assessment of cigarette smoking and questions regarding a family history of PD. All participants also completed the Mini-mental State examination[10], and a 20% sub-sample were selected for further assessment at baseline (A0) including those participants with obvious cognitive impairment. Participants were followed up after 2 years for a further structured interview (S2), or combined interview and assessment (C2). Informant data regarding PD status (H2) were also sought at follow-up. This study was approved by the Local Research Ethics committee, the details are available on the CFAS website[11].

### Definition of prevalent PD patient

During the initial screening phase of the study (S0), participants were asked the question- "Have you ever been diagnosed with PD?" All questions were asked by lay interviewers given standardised interview training for the purposes of this study, but without clinical experience of PD. These data therefore rely on self-reporting of PD status by study participants. We have Yes/No information on 12,652 of the 13,004 participants, which will be used as the denominator figure for prevalence estimates.

### Missing data and sensitivity analysis for PD prevalence estimate

Patients with missing data represent an important subgroup at this stage. We have missing data for 352/13004 participants, the reasons for which have been coded as follows-"No Answer" (n = 8), "Not asked" (n = 4), "Data Missing" (n = 340). Patients with missing data for this question include a high proportion of those with cognitive impairment, therefore we were concerned that this group may include a disproportionately high number of PD patients.

The (A0) assessment was performed on those patients identified as having cognitive impairment at the prevalence screen (S0) and a random sample of normal subjects (n = 2640). This number includes 32 patients self-reporting as suffering with PD as well as 234/352 of the patients with missing data regarding PD diagnosis. This assessment includes a section in which the lay interviewer rates the participant for several possible clinical signs of PD- "Slower Physically?", "Slow Movements?", "Expressionless face?", "Parkinsonian Movements", "Monotonous Voice?", and "Slow, Shuffling steps?" on a scale of "Not present, Mild or Severe".

Using data from those individuals with complete Yes/No data at S0 for the question "Ever diagnosed with PD", and also included in the A0 assessment, we constructed logistic regression models using Microsoft Stata to assess the usefulness of these assessment variables in predicting self-reported PD status. The best logistic regression model was chosen based on maximising -model sensitivity + 2 × model specificity, (greater emphasis placed on specificity to minimise false positive diagnoses). This model was fitted to those individuals with missing data from the prevalence screen (S0), and who were included in the assessment phase (A0), and the probability of their case status evaluated.

### The incidence of PD over 2 years

At the 2-year follow up interview, participants were asked the question "Have you been diagnosed with PD in the last 2 years?" An individual was considered an incident case of PD if they,

1.Denied PD at S0- AND

2.Admitted PD at S2 (n = 24) -OR

3.Admitted PD at C2 (n = 7)- OR

4.Informant stated PD at H2, with subject information (S2/C2) missing (n = 4)- OR

5.Information missing at S0 but "PD within 2 years at S2/C2" (n = 0)- OR

6.Information missing at S0 & S2/C2 but "PD within 2 years" from informant (H2) (n = 0).

The total number of person years at risk (denominator) was calculated for all individuals reaching the incidence stage with complete data for PD status, who had not been diagnosed with PD at S0. 4178 individuals (32%) were lost to follow up from the study between the S0 and S2/C2 stages, 1369 due to death (11%), 2621 refusal (20%) and 144 moved away (1%). We do not know the exact time point of the refusals or moved away groups hence these individuals were censored, and except for the small number of prevalent cases, we are unable to verify their PD status. These individuals are not part of the numerator or denominator for our PD incidence estimate.

The incidence of PD per 100 000 per year was calculated for the 5 different regions in the study. Cambridgeshire and Gwynedd can be considered predominantly rural communities, in comparison with Newcastle, Oxford and Nottingham, which represent more urban communities.

## Results

The overall prevalence of PD identified within this study for people aged over 65 years was 133/12652 = 1.05% (CI 0.87–1.2%). The age and sex specific figures for the prevalence of PD are presented in Tables 1 and 2, finding an increasing prevalence of PD with increasing age as expected, with consistently higher rates in men. There was no significant difference in rates between rural and urban communities. Age standardisation to the European 1991 population over the age of 65 years produces a prevalence rate of 0.95% (CI 0.67–1.23%).

Using our sensitivity analysis, we sought to evaluate the likely PD case status of 234 of the 352 patients with missing data at S0, and who had been assessed at A0. By maximising both sensitivity and specificity for PD case status in our logistic regression models we were able to conclude that 110/234 patients were very unlikely to have PD. Only 5/234 people were likely to be PD cases based on this analysis. If we include this additional information in our prevalence estimates, the overall prevalence increases to 138/12767 = 1.08% (CI 0.90–1.26%). Multi-variable adjusted Odds Ratios for some of the putative risks for PD among these self reported prevalent patients were calculated using logistic regression and are presented in Table 3.

### Incidence

Thirty five patients met our criteria for a self-reported incident PD case. The total number of person years at risk was 17,490, therefore our incidence estimate for self-reported PD was 200/100 000/yr, CI (144,278). Standardisation of the age-specific rates to the European 1991 population, produced an age-standardised incidence rate among the over 65s of 185/100 000/yr (95% CI 105–333).

There are small numbers of incident patients in each age and sex subgroup therefore the confidence intervals for these estimates are wide particularly in the very oldest age group (Table 4 and 5). Nevertheless, there appears to be a trend for increasing incidence of PD with advancing age and in men. There was no significant difference in incidence rates between the rural and urban regions.

All 5 of the patients thought to be "probable" prevalent PD cases died prior to the incidence screen. Multi-variable adjusted Odds Ratios for the same putative risks for PD among these self reported incident patients are presented in Table 6.

### Death certification and neuropathological diagnosis of patients self-reporting PD

At the time of this analysis, 6415 participants were deceased. 109/133 self-reported prevalent PD patients were deceased. The number with PD recorded on either part 1 or part 2 of their death certificates in this study was 44/109 (40%). Of the incident PD cases, 23/35 had died at the time of this analysis. The number with PD recorded on their death certificates was 8/23 (35%). 57 participants who had not been identified as diagnosed with PD at either the incident or prevalence screen also had PD recorded on their death certificates (57/6282 = 0.9%). We are unable to discriminate between these patients in whom the diagnosis of PD was made after the incidence screen and patients who had been diagnosed but did not self-report PD diagnosis. Of these 57 individuals the median time to death after S0 was 5.3 years (range 0.4–9.4 years), and among 24/57 who also participated in S2, the median time from S2 to death was 4.4 years (range 0.5–7.4 years). Approximately 25% of these individuals with death certificate diagnosis of PD, died within 2 years of a negative self report for PD.

Post mortem neuropathological examinations have been performed on a representative sample of participants in this study, including 10 brains from our patients self-reporting PD. Four of these brains showed typical features of PD, one brain had "insufficient Lewy bodies to make a diagnosis of PD", and there was one each of -progressive supranuclear palsy, Huntington's disease, basal ganglia mineralisation, cerebral amyloid angiopathy, and possible Alzheimer's disease. There were a further four individuals who had a neuropathological diagnosis of PD out of a total of 339 brains examined from individuals who did not report PD during the interview phases. The median time between their last negative PD self report and death was 3.0 years (range 0.8 years to 4.3 years).

## Discussion

The frequency figures reported here have been based on self-reported diagnosis of PD among a large population based cohort of elderly people within the UK. This method of detecting cases can potentially identify a greater percentage of medically diagnosed patients than using clinic or hospital records, since cases diagnosed within the primary care setting and not referred for specialist opinion will not be missed. However, patients that have not sought medical attention, or remain medically undiagnosed can only be reliably identified in multi-stage population screening studies. The S0 phase of the CFA study was not designed to identify medically undiagnosed PD cases, which therefore do not contribute to our prevalence and incidence estimates. Our sensitivity analysis suggests however that large numbers of obvious prevalent cases of PD have not been over-looked, although assessments were performed by lay interviewers rather than movement disorders specialists.

Acknowledging the possible non-identification of medically undiagnosed cases, our overall age standardised prevalence figure of 0.95% can be compared to the overall prevalence figure for PD of 1.6%, or for "parkinsonism" of 2.3%, identified in the EUROPARKINSON study of over 65s[6], that did use 2-phase screening methodology. Our data confirm the increased prevalence of PD into the highest age groups and in men. Our age standardised incidence figure for PD of 185/100 000 per year among over 65s, is comparable to the incidence figure of 250/100 000 per year among over 55s produced in the Rotterdam study[12] that also used 2 stage screening methodology.

At the time of the incidence screen (S2), 11% of this elderly cohort had died, and 20% refused to participate further, and among these individuals we are unable to quantify the number of cases of PD. Since PD symptoms leading to physical or cognitive decline may be more frequent among patients refusing participation, and are associated with premature mortality, we acknowledge that our incidence figure may be an under-estimate of the true incidence of PD in this cohort.

In comparison to cases examined by movement disorders specialists or investigated with functional imaging, self-reporting of PD diagnoses is also likely to result in a lowering of diagnostic accuracy. Clinical diagnoses of PD are only correct in 76–85% of cases even following assessment by movement disorders specialists[9], and diagnoses made within primary care will likely have even lower validity[13]. Neuropathological diagnosis of PD is considered the gold standard method of diagnosing PD although there have hitherto been no population based descriptive studies of the disease that include any neuropathological confirmation of disease status. The limited post-mortem data that we present in this study indicates that self-reported PD status acts as a more useful guide to presence of basal ganglia neuropathology rather than specific PD pathology. It is acknowledged therefore that the loss of diagnostic specificity in population-based studies such as this may impact on the analyses of potential risk factors.

Phillips et al[14] have previously confirmed the incomplete reporting of PD on death certificates, finding that only 76% of PD cases who had had diagnoses confirmed by neurologists had PD recorded in either Part 1 or 2 on their death certificates. Pressley found that 55% of patients had PD recorded on death certificates and there was a dependence on social class[15]. In our population study, it is not surprising that the percentage falls further, since diagnoses made in primary care may not be available for patients dying from unrelated causes in hospital, and so PD diagnosis may not be completed on the certificate. We did not observe any alternative diagnoses that might have caused extra-pyramidal symptoms, on the death certificates of our patients. This study therefore emphasises the inadequacy of only using routine mortality data in estimating disease frequency.

Analysis of several previously identified risk factors for PD among our prevalent cases confirms previous associations such as advancing age and male gender. A positive family history of the disease is however the largest and most significant risk factor (adjusted O.R. 2.5, p < 0.001). Inaccurate self-reporting of diagnoses usually tends to dilute rather than inflate observed risk factors for disease although in this instance, individuals with a family history of PD might be at greater risk of false positive diagnoses. A family history of PD has been previously associated with younger onset forms of the disease and our prevalent patients may include a small number with young onset forms of the disease. Our incident patients however represent a group of definite late onset patients in whom we have collected "risk" data prior to their onset of disease, which therefore should also be free from the effects of differential recall bias. Among our incident cases there is still an elevated OR for developing PD among people with a positive family history of disease, although the small numbers result in wider confidence intervals.

The results from this study give detailed frequency figures for the incidence and prevalence of self reported PD among a community-based population within the UK. The study lends some epidemiological based support for investigators searching for familial factors that increase the risk of the common late onset forms of the disease, but more importantly provides insight into the substantially lower reliability of self-reported PD diagnosis in comparison to the findings at post mortem examination.

## Competing interests

The author(s) declare that they have no competing interests.

## Authors' contributions

TF participated in the design of the study, carried out statistical analysis, and drafted the manuscript. CB, FM and LI participated in the design of the study, data analysis methods and critical revision of the manuscript. All authors read and approved the final manuscript.

## Pre-publication history

The pre-publication history for this paper can be accessed here:



## Figures and Tables

**Table 1:** Age and sex specific prevalence of self-reported PD in the CFAS study.

**Age Group**	**65–74**	**75–84**	**85+**	**Total**
**Male**	20/2808	33/1905	7/357	60/5070
	0.7%	1.7%	2.0%	1.2%
	CI 0.4–1.0	CI 1.1–2.3	CI 0.5–3.4	CI 0.9–1.5
**Female**	22/3486	36/3115	15/981	73/7582
	0.6%	1.2%	1.5%	1.0%
	CI 0.4–0.9	CI 0.8–1.5	CI 0.8–2.3	CI 0.7–1.2
**All**	42/6294	69/5020	22/1338	133/12652
	0.7%	1.4%	1.6%	1.1%
	CI 0.5–0.9	CI 1.1–1.7	CI 1.0–2.3	CI 0.9–1.2

**Table 2:** Prevalence of self reported PD in each of the 5 CFAS centres.

	**Cambridge**	**Gwynedd**	**Newcastle**	**Nott'ham**	**Oxford**	**Total**
**Male**	14/1098	8/1042	9/909	17/988	12/1033	60/5070
	1.3%	0.8%	1.0%	1.7%	1.2%	1.2%
	CI 0.6–1.9	CI 0.2–1.3	CI 0.4–1.6	CI 0.9–2.5	CI 0.5–1.8	CI 0.9–1.5
**Female**	10/1464	17/1509	14/1535	14/1452	18/1622	73/7582
	0.7%	1.1%	0.9%	1.0%	1.1%	1.0%
	CI 0.3–1.1	CI 0.6–1.7	CI 0.4–1.4	CI 0.5–1.5	CI 0.6–1.6	CI 0.7–1.2
**All**	24/2562	25/2551	23/2444	31/2440	30/2655	133/12652
	0.9%	1.0%	0.9%	1.3%	1.1%	1.1%
	CI 0.6–1.3	CI 0.6–1.4	CI 0.6–1.3	CI 0.8–1.7	CI 0.7–1.5	CI 0.9–1.2

**Table 3:** Analysis of risk factors for developing self-reported PD among prevalent patients.

	**Odds Ratio (OR)**		Multi-variable* adjusted OR	
	
	**OR(95% CI)**	**p value**	**OR (95% CI)**	**p value**
Age group (per 10 years)	1.64 (1.29–2.08)	<0.001	1.66 (1.29–2.13)	<0.001
Male sex	1.23 (0.87–1.74)	0.23	1.53 (1.04–2.23)	0.029
Family history of PD	2.73 (1.69–4.42),	<0.001	2.51 (1.50–4.20)	<0.001
Current smoking	0.39 (0.21–0.72)	0.003	0.45 (0.24–0.85)	0.01
Ever smoking	1.00 (0.70–1.45)	0.97	1.32 (0.85–2.05)	0.21
Cigarette dose- per 10 pack-years	0.90 (0.82–0.99)	0.03	0.89 (0.80–0.98)	0.023

*Adjusted ORs are presented for the multivariable logistic regression model which includes all listed risk factors. (Only cigarette dose was included as the smoking covariate except in the evaluation of "current smoking" and "ever smoking".)

**Table 4:** Age and sex specific incidence of PD per 100 000 per year in the CFAS study.

**Age Group**	**65–74**	**75–84**	**85+**	**All**
**Male**	191/100 000/yr	351/100 000/yr	527/100 000/yr	266/100 000/yr
	CI 96–382	CI 183–675	CI 132–2107	CI 170–417
	(8 cases)	(9 cases)	(2 cases)	(19 cases)
**Female**	78/100 000/yr	238/100 000/yr	194/100 000/yr	155/100 000/yr
	CI 29–208	CI 128–442	CI 49–776	CI 95–253
	(4 cases)	(10 cases)	(2 cases)	(16 cases)
**All**	129/100 000/yr	281/100 000/yr	284/100 000/yr	200/100 000/yr
	CI 73–227	CI 179–440	CI 107–756	CI 144–278
	(12 cases)	(19 cases)	(4 cases)	(35 cases)

**Table 5:** Age and sex specific incidence of PD per 100 000 per year in the CFAS study in each of the 5 CFAS centres.

**Cambridge**	**Gwynedd**	**Newcastle**	**Nottingham**	**Oxford**	**All**
173/100	216/100	209/100	238/100	172/100	200/100
000/yr	000/yr	000/yr	000/yr	000/yr	000/yr
(78–385)	CI 103–453	CI 100–438	CI 119–476	CI 82–360	CI 144–278
(6 cases)	(7 cases)	(7 cases)	(8 cases)	(7 cases)	(35 cases)

**Table 6:** Analysis of risk factors for subsequent development of self-reported PD among incident patients in CFAS.

	**Odds Ratio (OR)**		Multi-variable* adjusted OR	
	
	**OR (95% CI)**	**p value**	**OR (95% CI)**	**p value**
Age group (per 10 years)	1.64 (1.02–2.64)	0.04	1.48 (0.89–2.48)	0.13
Male sex	1.71 (0.88–3.34)	0.11	2.54 (1.21–5.35)	0.013
Family History of PD	1.91 (0.64–5.71)	0.25	1.86 (0.63–5.48)	0.26
Current smoking	0.93 (0.39–2.20)	0.87	1.12 (0.45–2.81)	0.80
Ever smoking	0.77 (0.39–1.52)	0.46	0.74 (0.32–1.76)	0.50
Cigarette smoking- per 10 pack-years	0.87 (0.72–1.06)	0.17	0.85 (0.70–1.05)	0.14

*Adjusted ORs are presented for the multivariable logistic regression model which including all listed risk factors.
